# Stability of Chimerism in Non-Obese Diabetic Mice Achieved By Rapid T Cell Depletion Is Associated With High Levels of Donor Cells Very Early After Transplant

**DOI:** 10.3389/fimmu.2018.00837

**Published:** 2018-04-24

**Authors:** Jiaxin Lin, William F. N. Chan, Louis Boon, Colin C. Anderson

**Affiliations:** ^1^Department of Surgery, University of Alberta, Edmonton, AB, Canada; ^2^Alberta Diabetes and Transplant Institutes, University of Alberta, Edmonton, AB, Canada; ^3^Bioceros BV, Utrecht, Netherlands; ^4^Department of Medical Microbiology and Immunology, University of Alberta, Edmonton, AB, Canada

**Keywords:** chimerism, tolerance, hematopoietic stem cell, transplantation, T cell depletion, non-obese diabetic mice

## Abstract

Stable mixed hematopoietic chimerism is a robust method for inducing donor-specific tolerance with the potential to prevent rejection of donor islets in recipients with autoimmune type-1 diabetes. However, with reduced intensity conditioning, fully allogeneic chimerism in a tolerance resistant autoimmune-prone non-obese diabetic (NOD) recipient has rarely been successful. In this setting, successful multilineage chimerism has required either partial major histocompatability complex matching, mega doses of bone marrow, or conditioning approaches that are not currently clinically feasible. Irradiation free protocols with moderate bone marrow doses have not generated full tolerance; donor skin grafts were rejected. We tested whether more efficient recipient T cell depletion would generate a more robust tolerance. We show that a combination of donor-specific transfusion-cyclophosphamide and multiple T cell depleting antibodies could induce stable high levels of fully allogeneic chimerism in NOD recipients. Less effective T cell depletion was associated with instability of chimerism. Stable chimeras appeared fully donor-specific tolerant, with clonal deletion of allospecific T cells and acceptance of donor skin grafts, while recovering substantial immunocompetence. The loss of chimerism months after transplant was significantly associated with a lower level of chimerism and donor T cells within the first 2 weeks after transplant. Thus, rapid and robust recipient T cell depletion allows for stable high levels of fully allogeneic chimerism and robust donor-specific tolerance in the stringent NOD model while using a clinically feasible protocol. In addition, these findings open the possibility of identifying recipients whose chimerism will later fail, stratifying patients for early intervention.

## Introduction

Mixed hematopoietic chimerism is the state of coexistence of donor- and recipient-derived hematopoietic cells in the host. Establishment of such chimerism, *via* allogeneic bone marrow transplantation (BMT), is a robust method for generating donor-specific tolerance to donor tissue/organs without the need for lifelong immunosuppression (1–7), and it can be used to treat severe autoimmune diseases (8, 9). However, its clinical application is dampened by the toxicity of current recipient conditioning regimens.

Although significant efforts have been made to generate reduced intensity and non-myeloablative conditioning protocols in murine models, the success of such protocols typically depends on the inclusion of total body irradiation (TBI), thymic irradiation, anti-CD40 ligand (anti-CD40L) monoclonal antibody (mAb), or a very high dose of bone marrow cells (BMC) (10–15). Of note, anti-CD40L mAb is known to cause thromboembolic complications in humans (16). A mega dose of BMC from one deceased donor is currently clinically unachievable (17), which would be relevant in the cases when cadaveric bone marrow and tissue/organs, such as islets, are the only option. Also, more stringent transplant settings, in which donor and recipient are fully major histocompatability complex (MHC) and minor histocompatability antigen (MiHA) mismatched, are often not tested.

More importantly, low-intensity conditioning protocols that induced mixed chimerism in C57BL/6 (B6) mice were not usually successful in autoimmune-prone, tolerance induction resistant recipients, such as non-obese diabetic (NOD) mice (18–20). The difficulty in inducing chimerism in NOD mice is manifested not only by a lower success of initial chimerism but also by the inability to maintain multilineage chimerism (21). In general, this obstacle in NOD mice can be overcome if irradiation (22–32), costimulation blockade (21, 25, 28, 30, 33–38), a high doses of rapamycin (21, 33–35, 38), or mega dose BMC (13, 15) from a fully MHC (13, 15, 21, 23, 24, 26, 30, 35, 38, 39) or more often partial MHC (22, 25, 27–29, 33–36) plus MiHA mismatched donor, are applied.

T cell depletion is another commonly used method for temporally inhibiting the host immune system. However, it was often used as adjuvant therapy with irradiation, costimulation blockade, or the combination of both (26, 28, 30, 32, 36–38). In a rare success, Zeng et al. induced fully mismatched chimerism in NOD mice conditioned with anti-CD3/CD8 and donor lymphocyte infusion (13, 15, 39). However, the transfer of a very high-dose BMC currently prevents the translation of this approach to a clinical setting. We previously showed that an irradiation-free mixed chimerism protocol in NOD mice is achievable with antibodies to T cells and CD40L together with busulfan (BUS) and high-dose rapamycin. We determined that recipient T cells were a critical barrier for generating chimerism in NOD recipients (38); however, the level of T cell depletion and its relationship to chimerism was not assessed. In addition, this protocol prevented donor islet rejection but did not generate tolerance to donor. Recently, we also developed a T cell depletion and rapamycin-based protocol that is irradiation and costimulation blockade free (40); however, donor chimerism waned over time.

Chimerism can be stable or transient in both animal models and in humans; and the loss of chimerism can increase the susceptibility of particular organs to rejection (41). The ability to identify early after BMT those recipients who will later lose chimerism would provide the opportunity to implement approaches that promote the stability of chimerism. We, therefore, sought to generate a more clinically feasible protocol fostering hematopoietic chimerism in stringent autoimmune-prone recipients and determine whether stability of chimerism is associated with events occurring early after BMT. We tested the hypothesis that maximizing recipient T cell depletion would eliminate the need for high-dose BMC or agents lacking clinical translatability (e.g., anti-CD40L and high-dose rapamycin) and would generate robust donor-specific tolerance across fully allogeneic barriers. We found that an extensive T cell depletion conditioning protocol, consisting of donor-specific transfusion (DST)-cyclophosphamide (CYP) and multiple T cell depleting antibodies achieved the goal of donor-specific tolerance and that very early levels of chimerism and donor T cells were significantly associated with the later stability of chimerism.

## Materials and Methods

### Animals

Adult NOD/ShiLtJ (H-2^g7^; termed NOD), FVB/NJ (H-2K^q^; termed FVB), C3H/HeJ (H-2K^k^; termed C3H), and B6.NOD-(D17Mit21-D17Mit10) (H-2^g7^; termed B6-g7) mice were purchased from the Jackson Laboratory (Bar Harbor, ME, USA), bred and housed in a specific pathogen-free facility at the University of Alberta. All care and handling of animals were conducted in accordance with the guidelines of the Canadian Council on Animal Care. All recipient mice used for chimerism induction were females at 8–10 weeks of age. MHC genotypes of the mice used are shown in Table S1 in Supplementary Material.

### Chimerism Induction Protocol and Definition of Chimerism and Health Status

Donor-specific transfusion with 20 × 10^6^ allogeneic splenocytes from the BMC donor strain was performed intraperitoneally (i.p.) on day −10 with respect to the date of BMT. CYP (Sigma, 150 mg/kg) was given on day −8 by a single i.p. injection. BUS (Sigma, 20 mg/kg) was administered i.p. on day −1. *In vivo* depletion mAbs were given as indicated in figure legends: anti-CD4 (GK1.5, 0.25 mg), anti-CD8α (53.6.7, 0.25 mg), anti-CD90.2 (30H12, 0.3 mg), and anti-asialo GM1 antibody (20 µL) from Wako Chemicals, USA. All mAbs were injected i.p. mAbs to CD4, CD8, and CD90 (anti-CD4/8/90.2) were purchased from Bio X Cell, West Lebanon, NH, USA, or generated by us. Allogeneic BMC (10–40 × 10^6^) were given intravenously *via* the lateral tail vein on day 0. Recipients were considered chimeric when at least 5% of MHC-I^+^ cells in the lymphocyte gate were donor derived at day 28 post-BMT. Stable chimerism was defined as the persistent presence of chimerism as assessed every 4 weeks post-BMT with the level of donor cells being no less than 20% of the level that was detected at day 28 post-BMT for at least 20 weeks. Body weight and blood glucose of recipient mice were monitored weekly. Mice with two consecutive blood glucose readings above 300 mg/dl were considered diabetic as assessed with a glucose meter (OneTouch, LifeScan, Canada).

### Antibodies and Flow Cytometry

Fluorochrome-labeled antibodies against H-2K^d^ (SF1-1.1.1), H-2K^k^ (36-7-5), H-2K^q^ (KH114), T-cell receptor (TCR) β (H57-597), CD4 (RM4-5 or RM4-4), CD8β (H35-17.2), CD11b (M1/70), CD11c (N418), B220 (RA3-6B2), CD49b (DX5), CD122 (TM-β1), FoxP3 (FJK-16s), Vβ11 (RR3-15), Vβ6 (RR4-7), and Vβ17a (KJ23) were purchased from BD Pharmingen (San Diego, CA, USA), BioLegend (San Diego, CA, USA), or eBioscience (San Diego, CA, USA). An LSR II (Becton Dickson, Sunnyvale, CA, USA) flow cytometer was used for data acquisition, and data analysis was performed using FlowJo VX (Treestar software, Portland, OR, USA).

### Skin Graft and Immunization Tests of Immunocompetence

Two pieces of 1 cm^2^ full thickness trunk skin from FVB and B6-g7 were transplanted onto the dorsum of recipient mice with 1 cm distance in between. The skin grafts were secured with sutures to the recipient graft bed and then bandaged for 7 days. The grafts were inspected daily and considered rejected at the time when approximately 90% surface area was necrotic.

As an additional test of immunocompetence, mice that had remained chimeric for 13–15 months were immunized with ovalbumin (OVA) and serum anti-OVA antibodies were assessed by ELISA. Stable chimeric FVB**→**NOD and naive NOD mice were immunized with 100 µL OVA/complete Freund’s adjuvant (CFA) containing 50 µL of 2 mg/mL OVA (Sigma-Aldrich, USA) and 50 µL CFA (OZ Biosciences, France) subcutaneously on the hind legs. Mice were bled *via* submandibular vein 21 days postimmunization and serum was stored at −80°C. To detect OVA-specific mouse IgG, 96-well flat bottom plates (Corning Inc., USA) were coated with 1 µg OVA in 100 µL 0.1 M sodium carbonate-bicarbonate buffer (pH 9.6) at room temperature for 2 h. After washing three times with washing buffer [0.05% Tween 20 in phosphate-buffered saline (PBS), pH 7.4], plates were blocked with assay buffer (1% FBS in PBS, pH 7.4; for 2 h) and then incubated for 2 h with 100 µL of twofold serial dilutions of serum in assay buffer. Plates were then washed three times and incubated for 1 h with 100 µL of 1:5000 dilution of Peroxidase AffiniPure Donkey Anti-Mouse IgG (715-035-150, Jackson ImmunoResearch, USA). Incubation steps were at room temperature with plates placed on a plate shaker. Plates were washed four times and incubated with 100 µL TMB substrate solution (OptEIA reagent set; BD) in the dark. After 10 min, 100 µL 0.16 M sulfuric acid was added, and the optical density (OD) at 450 nm was measured using an ELISA plate reader (μQuant Microplate Spectrophotometer and Gen5, Bio-Tek, USA). OD values from the duplicate wells were averaged, and OD values of negative control wells (OD~0.05) that were not cultured with serum were subtracted to remove background. The average OD value of control wells that were not coated with OVA but had serum added (1/10,000 dilution) from each sample was approximately 0.05.

### Statistical Analysis

One-way ANOVA with Tukey’s multiple comparison test, Student’s *t*-test with Welch’s correction when variances were significantly different were used accordingly. All statistical analyses were done using Prism 7 (GraphPad Software, San Diego, CA, USA) with statistical significance defined as *p* < 0.05.

## Results

### DST-CYP Preferentially Prevents the Expansion of Alloreactive Host T Cells in NOD Mice

**Figure 1:**
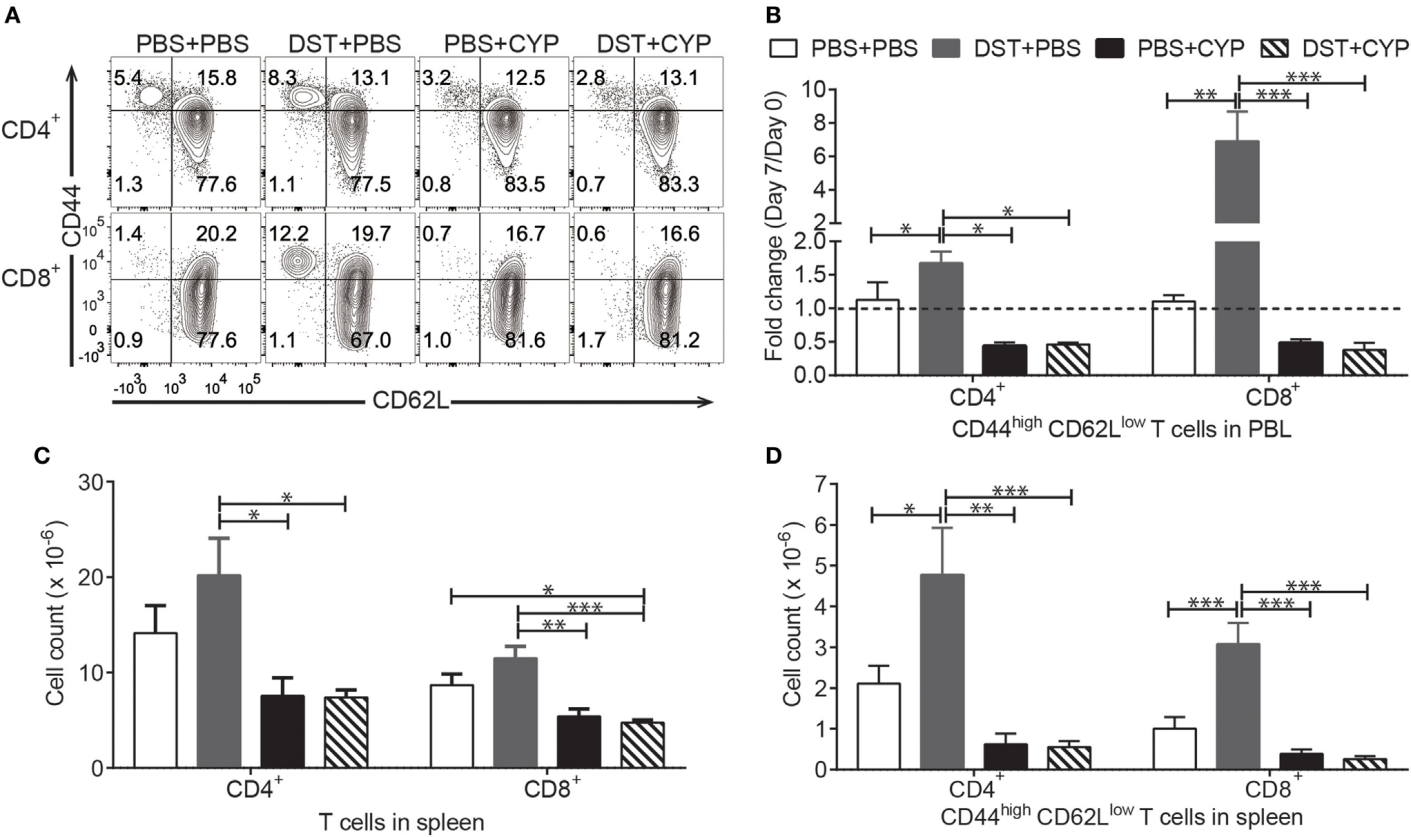
Donor-specific transfusion (DST)-cyclophosphamide (CYP) is effective in autoimmune recipients. Naive female non-obese diabetic (NOD) mice were given 20 × 10^6^ C3H splenocytes or vehicle [phosphate-buffered saline (PBS)] intraperitoneal (i.p.) on day 0 and a dose of CYP, or vehicle i.p. on day 2. Peripheral blood lymphocytes (PBL) were taken on day 0 and day 7 for analysis. All mice were euthanized on day 7 and splenocytes were harvested for analysis. **(A)** Representative analysis of PBL on day 7, CD4^+^TCRβ^+^ gate for the upper panel and CD8^+^TCRβ^+^ gate for the bottom panel. **(B)** Shown is fold change of CD44^high^ CD62L^low^ cells in CD4^+^TCRβ^+^ gate (left) and CD8^+^TCRβ^+^ gate (right) on day 7 compared to day 0 (mean ± SEM). **(C)** Shown are absolute numbers of CD4 T cells (left) and CD8 T cells (right) in the spleen on day 7 (mean ± SEM). **(D)** Shown are absolute numbers of CD44^high^ CD62L^low^ CD4 T cells (left) and CD44^high^ CD62L^low^ CD8 T cells (right) in the spleen on day 7 (mean ± SEM). Data were pooled from four independent experiments (four to six mice per group). *p < 0.05, **p < 0.005, ***p < 0.001, one-way ANOVA with Tukey’s multiple comparison test.

To create an efficient T cell depletion based conditioning protocol, we employed DST-CYP with T cell depleting mAbs. CYP administration following DST is also called cells-followed-by-CYP system, which is proposed to eliminate actively dividing alloreactive T cells (42). Although this system has been widely shown to be valid in different strains of mice, it was rarely tested in the tolerance resistant NOD mouse model of type-1 diabetes, especially for chimerism induction (27, 34, 40). Lee et al. showed that DST-CYP along with regulatory T cell (Treg) transfusion prolonged the survival of allogeneic islets in autoimmune diabetic mice. However, it appeared that DST-CYP depleted alloreactivity in a non-specific fashion (43). Whether DST-CYP could preferentially block the generation of allostimulated effector cells in NOD mice has not been reported. Here we examined this by conditioning naive NOD mice with vehicle, DST-vehicle, vehicle-CYP, or DST-CYP and comparing the frequencies of effector memory (CD44^high^ CD62L^low^) T cells in peripheral blood lymphocytes (PBL) before and after treatment (Figure 1A). Preconditioning with fully allogeneic splenocytes alone led to an over 1.5-fold expansion of effector memory CD4 T cells and sevenfold expansion of effector memory CD8 T cells in PBL (Figure 1B). In contrast, CYP alone reduced the frequency of effector memory CD4 and CD8 T cells by approximately 50% in PBL, while DST-CYP completely prevented the expansion of effector memory cells caused by the DST (Figure 1B). Results from splenocyte analysis on day 7 showed that DST alone tended to increase the absolute numbers of CD4 and CD8 T cells, and significantly increased the numbers of effector memory CD4 and CD8 T cells compared to the vehicle treated group (Figures 1C,D). DST-CYP prevented this increase and significantly reduced the absolute numbers of CD4 and CD8 T cells as well as their effector memory subsets (Figures 1C,D). In addition, NOD mice treated with CYP alone showed a trend towards reduction of T cells and effector memory T cells (not statistically significant; Figures 1C,D). Thus, while DST-CYP causes some generalized T cell depletion, it preferentially and effectively prevents the expansion of allostimulated effector memory T cells in NOD mice.

### Combining of DST-CYP and Dual Anti-T Cell mAb Treatment Peri-BMT Induces Chimerism That Lacks Stability and Is Donor Dependent

**Figure 2:**
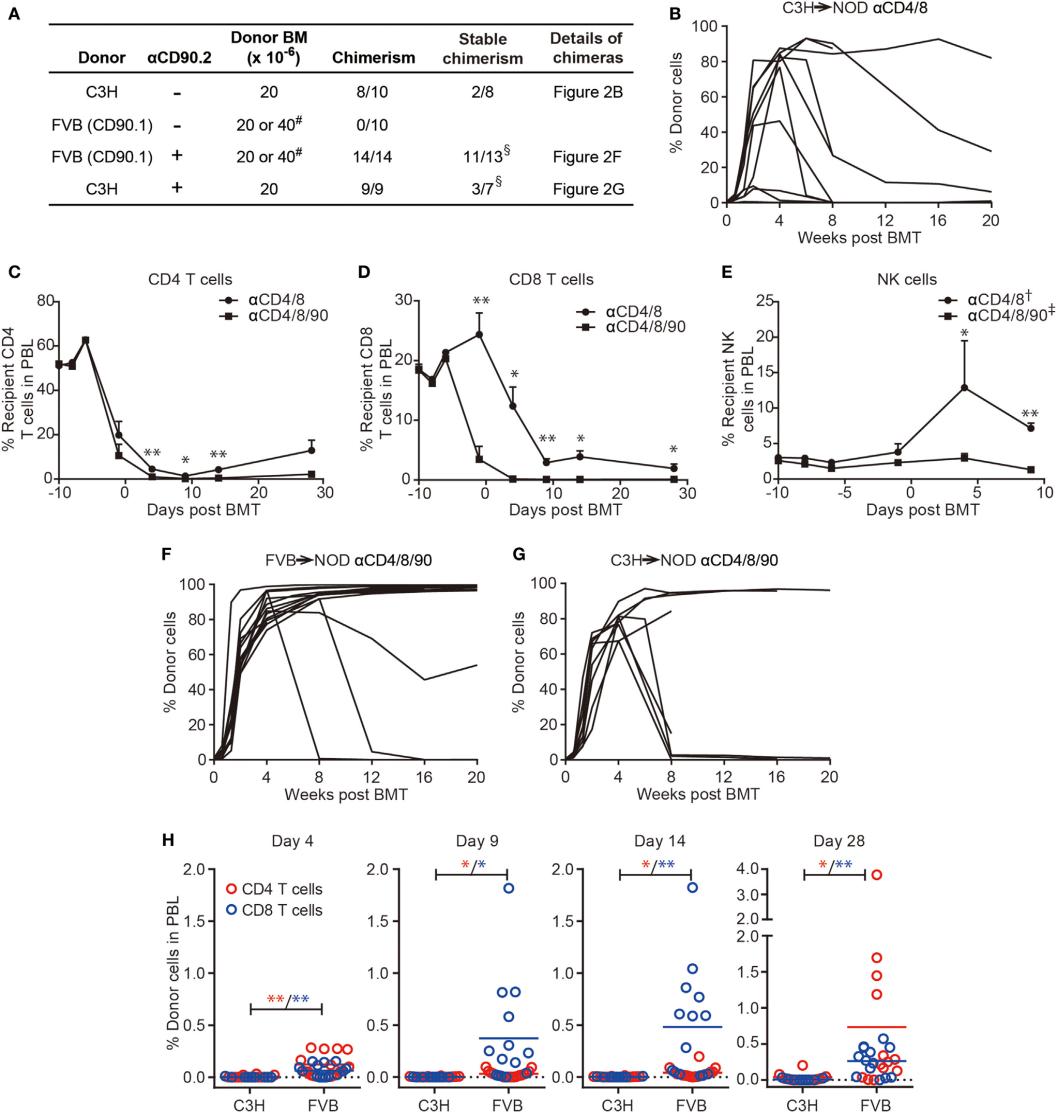
Robust T cell depletion in non-obese diabetic (NOD) mice preconditioned with donor-specific transfusion (DST)-cyclophosphamide (CYP) allows chimerism using fully allogeneic donor hematopoietic cells. **(A)** NOD recipients were conditioned with DST (day −10), CYP (day −8), a combination of T cell depletion monoclonal antibodies (mAbs) (anti-CD4/8 ± anti-CD90, days −6, −1, 4, 9, 14; “-” indicates no injection), busulfan (day −1), and bone marrow cells (BMC) (day 0, 20, or 40 × 10^6^). # two recipients from each group given 40 × 10^6^ BMC, the remainder received 20 × 10^6^; cells were from the same donor strain as the DST. The success of chimerism was determined at 4 weeks postbone marrow transplantation. ^§^One FVB→NOD and two C3H→NOD chimeras were excluded as they were found dead prior to analysis of the stability of chimerism. **(B,F,G)** Shown are the proportions of donor cells in lymphocyte gate in peripheral blood lymphocytes (PBL) over time. **(C–E)** PBL were harvested before each treatment to evaluate the CD4, CD8 T cell, and NK cell components in FVB→NOD recipients conditioned with anti-CD4/8 or anti-CD4/8/90 mAbs. Shown are percentages of recipient CD4, CD8 T cell, and NK cells in the lymphocyte gate (mean ± SEM). NOD recipients were treated with anti-CD4/8 (*n* = 10; † *n* = 2) or anti-CD4/8/90 (*n* = 14; ‡ *n* = 6) mAbs. **(H)** PBL were harvested at the indicated time points to evaluate the donor CD4 and CD8 T cells in C3H→NOD or FVB→NOD recipients conditioned with anti-CD4/8/90 mAbs. Shown are percentages of donor CD4 and CD8 T cell in the lymphocyte gate (mean). Data were pooled from at least two independent experiments. *p < 0.05, **p < 0.005, Student’s *t*-test with Welch’s correction when variances were significantly different.

We then asked if the combination of DST-CYP and T cell deple-ting mAbs induces chimerism in NOD mice by using fully allogeneic donors. NOD mice were preconditioned with DST from C3H mice, CYP, antibodies against CD4/8, BUS, and a donor bone marrow transplant. By using this protocol, mixed chimerism was induced in 8 of 10 NOD mice with six chimeras having levels of chimerism higher than 75% at 4 weeks post-BMT (Figure 2). Despite the high-level chimerism at 4 weeks post-BMT, only two chimeras were able to maintain substantial chimerism long-term (Figure 2B) with multiple-lineages of donor cells, including T, B, NK, and dendritic cells (data not shown). Four chimeras quickly lost their chimerism at 8 weeks post-BMT. Although we found no obvious signs of graft-versus-host disease (GVHD), such as chronic weight loss and dermatitis, or hyperglycemia in chimeric recipients, two recipients died at 9 and 14 weeks post-BMT. However, these results already strongly supported the hypothesis that by employing DST-CYP and T cell depleting mAbs mixed chimerism could be induced in NOD mice without irradiation, costimulation blockade or rapamycin.

As C3H only represents one fully allogeneic donor, we sought to test this protocol with a second fully allogeneic donor, FVB, to test the stringency of the current protocol. Donor and recipient MHC disparities are shown in Table S1 in Supplementary Material. Surprisingly, none of the 10 NOD mice became chimeric, even in the 2 recipients given a double dose of BMC in a single injection (Figure 2A row 2). Thus, the combination of DST-CYP and anti-CD4/8 mAbs induced multilineage chimerism when using a C3H donor. However, this success could not be extended to the FVB→NOD combination.

### A Triple Anti-T Cell mAb Protocol Facilitates the Depletion of Recipient T and NK Cells and the Induction of High-Level Chimerism

As CD8 T cells (44) and NK cells (45) are both important barriers to chimerism induction, we hypothesized more efficient CD8 T and NK cell depletion would prevent bone marrow rejection in the FVB to NOD combination and induce chimerism. We included anti-CD90 mAbs in the new protocol, as CD90 is expressed not only on T cells but also a subset of NK cells (46). As shown in Figure 2C, the combination of anti-CD4/8/90 mAbs (termed triple antibody protocol) modestly, although significantly, increased depletion of CD4 T cells compared to anti-CD4/8 mAbs (termed duo antibody protocol). More strikingly, the triple antibody protocol accelerated the depletion of CD8 T cells compared to the duo antibody protocol (Figure 2D). The superiority of the triple antibody protocol in NK cell depletion was not obvious until the infusion of the third dose of antibodies compared to the duo antibody protocol (Figure 2E).

**Figure 3:**
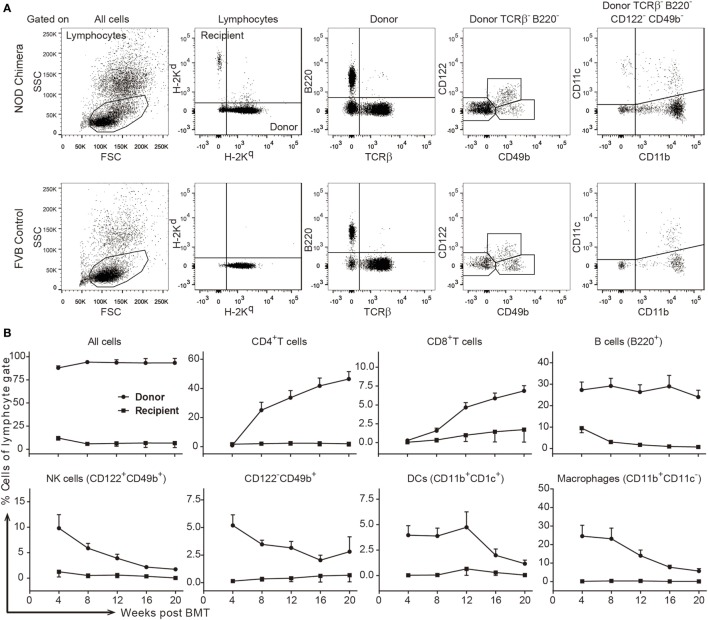
Stable high-level multilineage donor chimerism was maintained in FVB→non-obese diabetic (NOD) chimeras conditioned with a robust T cell depletion protocol. Stable chimeras (FVB→NOD; *n* = 11) induced by the triple antibody protocol (refer to Figure 2B) were analyzed for different lineages of donor- and recipient-derived cells in peripheral blood lymphocytes (PBL) over time. **(A)** Shown is the gating strategy. **(B)** Shown are the percentages of donor- or recipient-derived major histocompatability complex-I^+^ cells, CD4^+^ T cells, CD8^+^ T cells, B cells, NK cells, TCRβ^−^B220^−^CD122^−^CD49b^+^ cells, DCs, and macrophages in the lymphocyte gate in PBL over time. Values are shown as the mean ± SEM.

With the success of host T and NK cell depletion after the inclusion of anti-CD90 mAbs, chimerism was induced in 14 of 14 NOD recipients using FVB BMC (Figure 2A row 3; Figure 2F). Though 1 mortality was found at 8 weeks post-BMT without obvious signs of GVHD, 11 of 13 remaining chimeras maintained stable multilineage chimerism (Figures 3A,B). These data were in agreement with our previous finding that NOD T cells are the major cells that mediate split tolerance in chimerism induction (38) and a robust T cell depletion based regimen could overcome split tolerance. Strikingly, 10 of the 11 stable chimeras developed nearly complete chimerism (Figures 2F and 3). The generation of complete chimerism is considered to be more difficult to achieve compared to mixed chimerism, with the establishment of a higher level of chimerism in NOD mice requiring a higher dose of BMC (24), a higher dose of irradiation (30), and more costimulation blockade (30) when fully allogeneic donor cells are used. Moreover, full chimerism had not previously been achieved in NOD mice conditioned with an irradiation free protocol and given fully allogeneic BMC (13, 15, 21, 35, 38, 40).

As shown in Figure 2B, only two NOD mice conditioned with the duo antibody protocol and infused with C3H BMC developed stable mixed chimerism. We asked if the success of the triple antibody protocol with FVB BMC could be applied to C3H BMC recipients. In this case, all nine NOD mice became chimeric at 4 weeks post-BMT when treated with the triple antibody protocol. However, the success of chimerism induction with anti-CD90 mAbs in C3H BMC recipients was not as striking as in FVB BMC recipients. On the one hand, the inclusion of anti-CD90 mAbs did not increase the rate of stable chimerism, as only three NOD mice became stable chimeras (Figures 2A,G) with multiple lineages of donor cells (data not shown). Four mice had unstable chimerism (chimerism declined by 8 weeks post-BMT; one was found dead at 11 weeks), and another two were found dead at 6 and 10 weeks post-BMT without obvious signs of GVHD (Figure 2G) prior to the determination of chimerism stability. On the other hand, targeting CD90 improved the levels of donor cells in stable C3H→NOD chimeras, as full chimerism was maintained in three C3H→NOD chimeras treated with the triple antibody protocol (Figure 2G). In contrast, full chimerism was not observed in any of the C3H→NOD chimeras treated with the duo antibody protocol (Figure 2B).

The success of BMT results not just from less rejection by recipient immune cells but also the promotion of BMC engraftment mediated by donor cells, within which donor CD8 T cellshave been shown to play a role (13, 47). Indeed, though recipient T cells, as well as NK cells, were depleted equally in both C3H→NOD and FVB→NOD chimeras, donor cells from FVB (CD90.1) were not susceptible to the anti-CD90.2 mAb we employed for depletion. We therefore asked if the difference of success in chimerism induction with FVB and C3H donors was associated with the presence of donor passenger T cells. We observed a significantly increased frequency of donor CD8 T cells in FVB→NOD but not C3H→NOD chimeras at early time points post-BMT (Figure 2H). Although this early existence of donor T cells was associated with the success of FVB→NOD chimerism generation, it was not an absolute requirement for the current protocol, as chimerism could in some cases be established even when donor passenger T cells were depleted in C3H→NOD chimeras. Taken together, the inclusion of anti-CD90 enhanced recipient T and NK cell depletion and greatly facilitated the induction of stable high-level chimerism using a donor that had a maximal class I mismatch (FVB). However, this facilitation was much less apparent when using a donor (C3H) that had a maximal class II mismatch and T cells susceptible to the anti-CD90. These findings are consistent with the major effect of anti-CD90 being a more efficacious depletion of CD8 T cells (Figure 2D).

### NOD Mice With High Level Chimerism Acquire Robust Donor-Specific Tolerance and Recover a Substantial But Diminished Level of Immunocompetence

**Figure 4:**
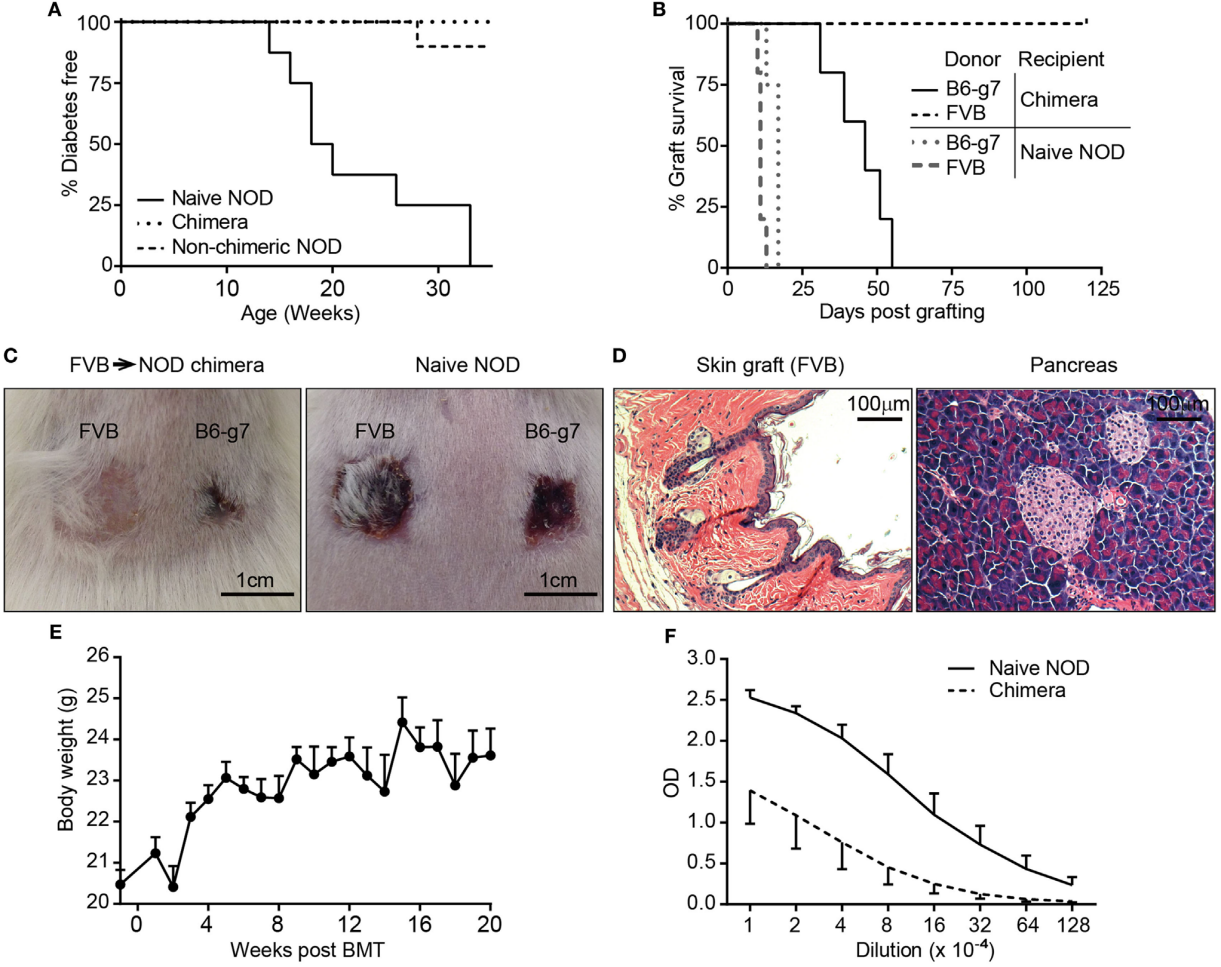
Chimeras with high level or complete chimerism acquired full donor-specific tolerance, did not develop autoimmune disease, and recovered immunocompetence. **(A)** Lack of autoimmune diabetes in chimeras. Recipients [C3H→ non-obese diabetic (NOD) and FVB→NOD; *n* = 43] conditioned with duo or triple antibody protocol (refer to Figure 2A) and naive female NOD mice were monitored for blood glucose weekly starting from 8 to 10 weeks of age. Shown are percentages of mice that were diabetes-free over time (chimeric mice, *n* = 31; non-chimeric mice, *n* = 12; naive NOD, *n* = 8). **(B)** FVB→NOD (*n* = 5) and Naive NOD mice (*n* = 5) were engrafted with skin from FVB and B6-g7 donors at 6 months after bone marrow transplantation (BMT). **(C)** Shown are representative macroscopic pictures of the acceptance and/or rejection of skin grafts in FVB→NOD chimeras (51 days postskin transplant) and naive NOD mice (13 days postskin transplant). **(D)** Pancreas and FVB donor skin (6 months postskin transplantation) from FVB→NOD chimeras that maintained donor chimerism for about 12 months were subjected to hematoxylin and eosin staining. Shown are representative photographs from individual chimeras (*n* = 4). **(E)** Shown is the body weight of FVB→NOD chimeras (refer to Figure 2F; mean ± SEM). **(F)** FVB→NOD (*n* = 4) and Naive NOD mice (*n* = 5) were immunized with ovalbumin (OVA) at 12 months after BMT. Serum was collected 3 weeks postimmunization and for anti-OVA IgG detection. Shown are optical density value (mean ± SEM) for each serum dilution.

After the success in generating stable multilineage chimerism, we sought to examine if NOD chimeras reestablished tolerance to self-antigens and displayed donor-specific transplantation tolerance. Chimerism induction with allogeneic BMC has been shown to reestablish tolerance to self-antigens in NOD mice. First, and consistent with previous studies (48), we found that the FVB→NOD chimeras remained free of diabetes and lacked islet infiltration (Figures 4A,D). In contrast, all naive NOD mice, and some NOD mice that were conditioned with a duo or triple antibody protocol without developing chimerism, became diabetic over time (Figure 4A). Second, the tolerance status in chimeras was bidirectional. On the one hand, the successful engraftment of donor hematopoietic cells represents tolerance to the donor antigens present in donor hematopoietic-derived cells. More importantly, FVB→NOD chimeras accepted FVB donor skin grafts indefinitely (Figures 4B–D), which represents the most stringent test of tolerance to a donor and indicates that tolerance extends beyond hematopoietic cells to other donor tissue antigens. And finally, their healthy appearance, and continued increase in body weight (Figure 4E), suggested a lack of GVHD in FVB→NOD chimeras with complete chimerism, indicating donor cells were tolerant of host antigens.

In addition to the tolerance status of chimeras, immunocompetence is another important concern, particularly in the case of full chimerism. Full chimerism has been considered by some to be less desirable as there is the potential the recipient will have some immunodeficiency due to the T cells being selected in a thymus that has different MHC alleles (recipient MHC type) than that on the antigen presenting cells (donor MHC type) (3, 49). To address if NOD mice with full chimerism were immunocompetent, FVB→NOD chimeras were also transplanted with skin from B6-g7 mice (third party), which has the MHC genes from NOD and the non-MHC genes from B6 [i.e., mismatched for multiple MiHA as well as gene(s) regulating innate allo-responses (50)]. All chimeric and naive NOD mice were able to reject B6-g7 skin (Figures 4B,C), although chimeras rejected B6-g7 skin more slowly. Chimeric NOD mice were also immunized with OVA to evaluate anti-OVA antibody production for determining the level of humoral immunocompetence. As shown in Figure 4F, NOD chimeras produced substantial IgG against OVA, although the titer that was approximately eightfold less than in young naive NOD mice. Together, these data indicate that the chimeras were fully tolerant of the donor with substantial but diminished immunocompetence.

**Figure 5:**
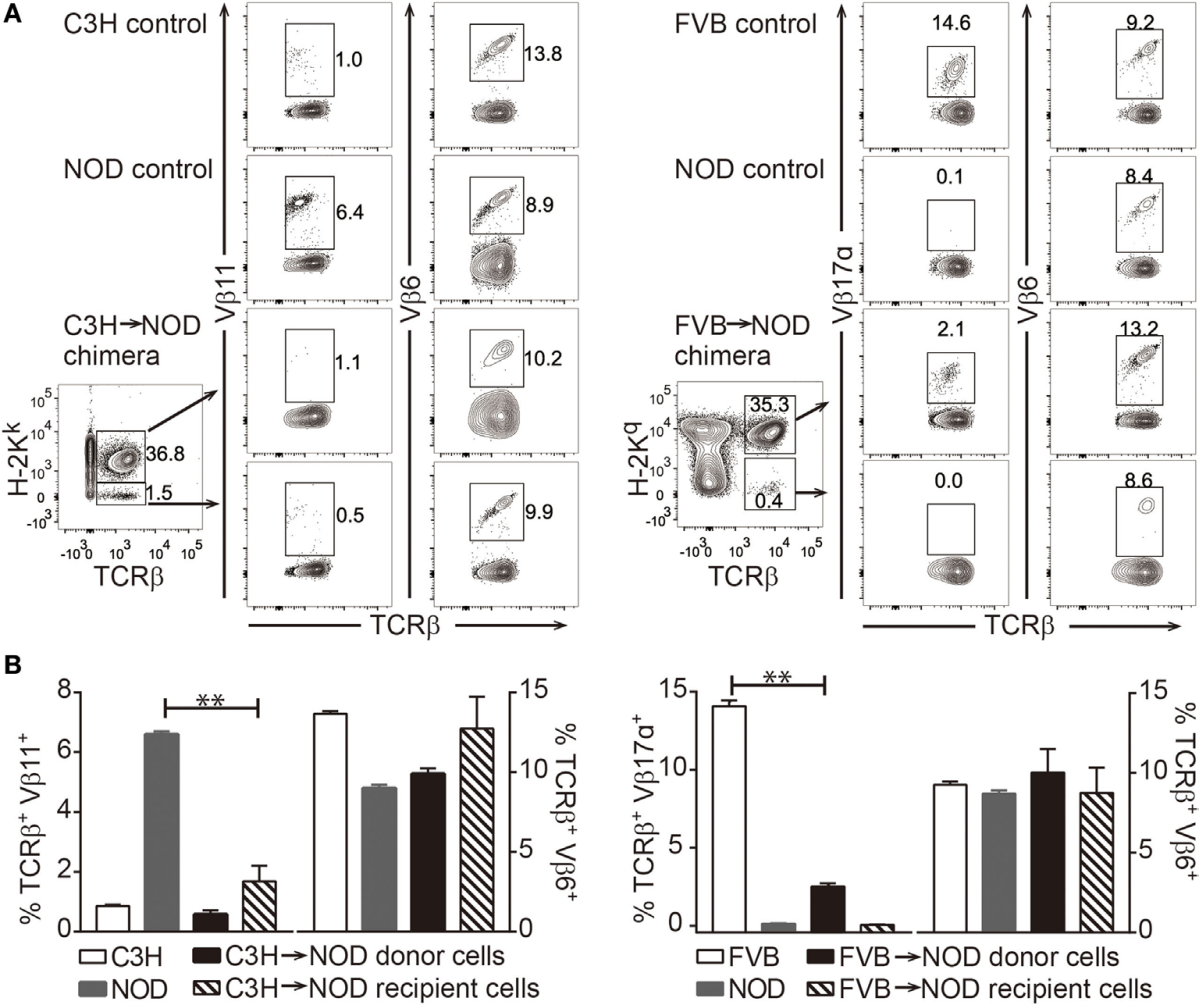
Clonal deletion of alloreactive T cells in chimeras. **(A,B)** Frequencies of recipient anti-donor (Vβ11^+^) T cells in C3H→non-obese diabetic (NOD) chimeras and donor antirecipient (Vβ17α^+^) T cells in FVB→NOD chimeras were evaluated at 8–12 weeks postbone marrow transplantation. **(A)** Shown is the gating strategy. **(B)** Shown are the frequencies of Vβ11^+^, Vβ6^+^, and Vβ17α^+^ T cells in the peripheral blood lymphocytes. Values are shown as the mean ± SEM. Numbers of animals used for analysis in the left panel: naive C3H *n* = 6, naive NOD *n* = 7, C3H→NOD chimeras *n* = 4; right panel: naive FVB *n* = 4, naive NOD *n* = 4, FVB→NOD chimeras *n* = 8. **p < 0.005, Student’s *t*-test with Welch’s correction when variances were significantly different.

As central tolerance *via* clonal deletion has been proposed as the main mechanism for donor-specific tolerance *via* chimerism induction, we investigated whether clonal deletion was occurring in the chimeras. Superantigens encoded by endogenous viral genes in mice are known to elicit strong binding of particular TCR variable-beta (Vβ) chains and the MHC class II molecule, which then leads to the deletion of certain Vβ^+^ T cells (51). This phenomenon “mimics” the process of clonal deletion during normal T cell development in the thymus. As a result, the frequency of Vβ11^+^ T cells in C3H and Vβ17a^+^ T cells in NOD mice are much lower compared to Vβ6^+^ T cells. In our model, the recipient Vβ11^+^ T cells bind to C3H-derived superantigen in C3H→NOD chimeras with the presence of I-E^k^ and donor Vβ17a^+^ T cells react with NOD-derived superantigen in FVB→NOD chimeras with the presence of I-A^g7^ (52–54). We found the reduction of these two populations occurred in the chimeras (Figures 5A,B) compared to naive controls, which indicated that clonal deletion of at least a subset of donor-reactive and host-reactive T cells had occurred.

### The Eventual Loss of Chimerism Is Associated With Lower Levels of Chimerism and Donor T Cells Early After BMT

**Figure 6:**
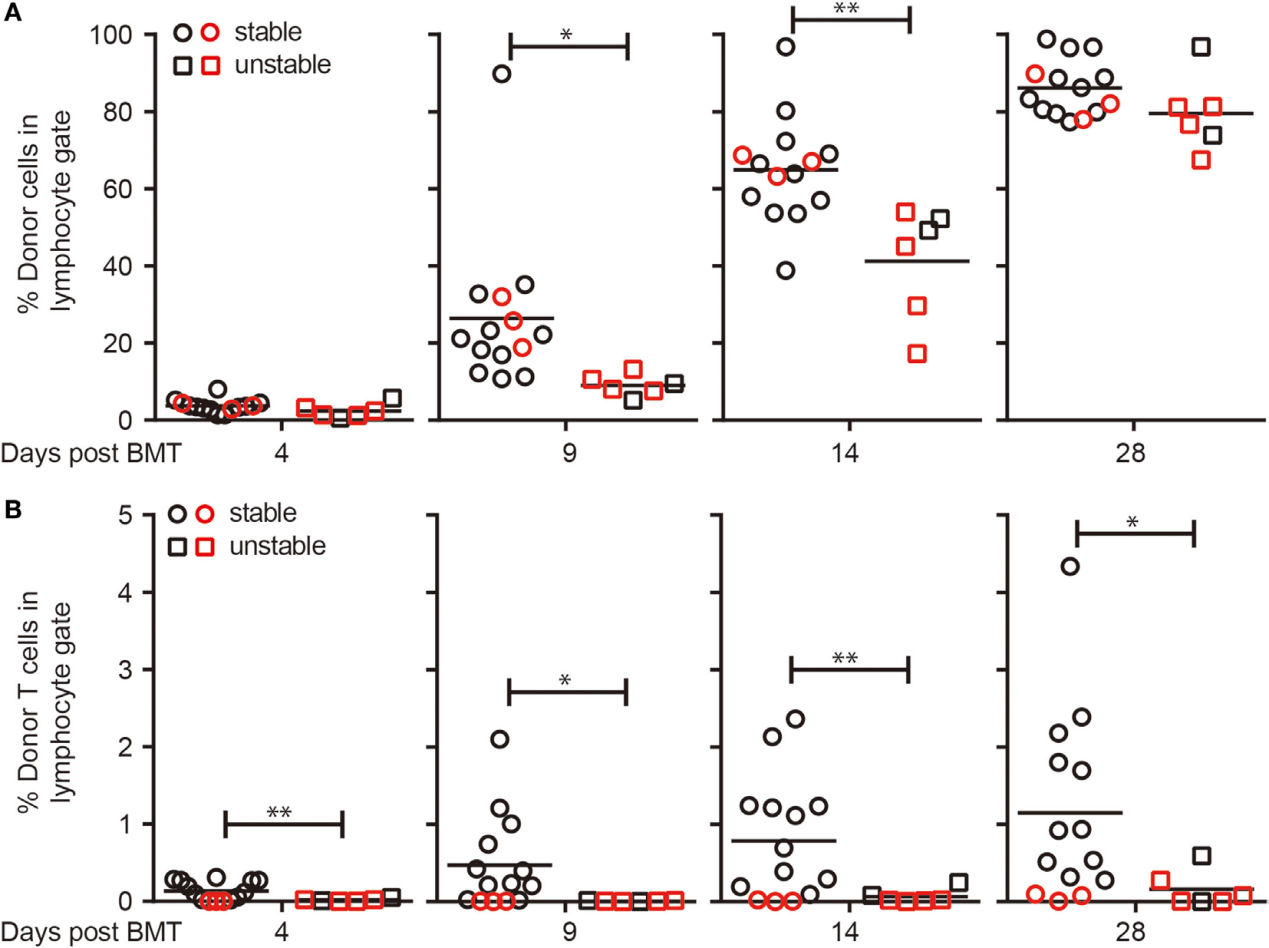
Loss of chimerism is significantly associated with lower early levels of chimerism and donor T cells. Non-obese diabetic (NOD) recipients were conditioned with donor-specific transfusion (DST) (day −10; using C3H or FVB splenocytes), cyclophosphamide (day −8), anti-CD4/8/90 monoclonal antibodies (day −6 and every 5 days until day 14), busulfan (day −1), and bone marrow cells (BMC) (day 0, 20 × 10^6^; using BMC from same donor strain as the DST). Peripheral blood lymphocytes (PBL) were harvested before each treatment to evaluate the donor cell components in the lymphocyte gate. Comparison of early levels of chimerism **(A)** and donor T cells **(B)** in recipients that maintained stable chimerism (*n* = 14) and those had unstable chimerism (*n* = 6). Black: FVB→NOD; red: C3H→NOD. *p < 0.05, **p < 0.005, Student’s *t*-test with Welch’s correction when variances were significantly different.

FVB→NOD and C3H→NOD chimeras treated with the triple antibody protocol developed high-level chimerism that was either maintained or the chimerism was lost between 6 and 12 weeks post-BMT (Figures 2F,G). Strikingly, the decrease of chimerism in these unstable chimeras was sudden and sharp despite the presence of a very high level of chimerism in the previous 2–4 weeks (Figures 2F,G). Being able to predict which recipients will lose chimerism later on would provide the opportunity for early interventions on a individual basis. We therefore sought to determine if there might be some intrinsic differences between the stable and unstable chimeras at early time points post-BMT that would be detectable and associated with the fate of chimerism in the long term. In an attempt to address this issue, we compared the chimerism and donor T cell levels at days 4, 9, 14, and 28 in mice that maintained stable chimerism to those whose chimerism level had dropped more than 80% from the level at day 28 (Figure 6). We found that chimeric NOD mice that maintained stable chimerism had significantly higher donor chimerism at days 9 and 14 post-BMT (Figure 6A) and this significant difference was also apparent when donor T cells were excluded from the analysis (data not shown). Higher levels of donor T cells at very early time points post-BMT (from day 4) were also found in stable chimeras compared to chimeric mice that would later lose their chimerism (Figure 6B). Thus, despite the continued rise of chimerism levels for a period and eventually exceeding 60% donor cells in all recipients, some recipients subsequently lost chimerism, and this was significantly associated with a lower level of chimerism and donor T cells very early after BMT.

## Discussion

Mixed hematopoietic chimerism is considered the most robust method for inducing donor-specific tolerance to prevent organ rejection. However, its clinical application has been impeded by the toxicity and complexity of current recipient conditioning regimens. We and others have been focusing on developing reduced intensity host condition protocols in murine models. However, several issues remain in the current conditioning protocols, which prevent the translation of these protocols into clinical applications. The limitations include the use of irradiation, high multiple doses of chemotherapeutics, and a mega dose of BMC as well as thrombogenic anti-CD40L mAb. Moreover, allogeneic chimerism induction is more difficult to achieve in tolerance defective recipients that develop autoimmune disease, such as diabetes-prone NOD mice, when compared to non-autoimmune strains (13, 23–25, 28). We showed here that a robust T cell depletion by an optimized DST-CYP and T cell depleting mAb combination leads to success in generating chimerism in NOD mice even when using a clinically relevant amount of fully allogeneic BMC, without the inclusion of irradiation, costimulation blockade, and rapamycin.

We demonstrated that the key factor in our current protocol is the administration of DST-CYP and anti-CD4/8/90 mAbs. First, we confirmed that DST-CYP led to the preferential inhibition of allostimulated T cells, which was likely due to the killing of these cycling cells. Moreover, NOD recipients conditioned with the triple antibody protocol without DST only developed transient chimerism, with lower levels of donor cells at 4 weeks post-BMT (Figures S1A,B in Supplementary Material). As the inhibition of donor-specific T cells is not sufficient without DST, the alloreactive naive and memory T cells that survived CYP treatment might be activated after BMT. Such alloreactive T cells, mainly CD4 T cells, then increased in frequency in lymphopenic hosts (Figure S1C in Supplementary Material) and rejected all the donor cells rapidly.

T cell depletion mediated by either monoclonal or polyclonal antibodies has been used for solid organ transplantation and hematopoietic chimerism induction for several decades (55). Although the employment of such antibodies could eliminate over 90% of T cells in the periphery in most cases, the depletion of T cells is less efficient in spleen, thymus, and tissues. In addition, memory T cells are resistant to antibody-mediated depletion compared to naive T cells (56). Moreover, T cell depletion creates a space and resource enriched microenvironment for residual T cells, which then undergo lymphopenia-induced proliferation (LIP) and are more likely acquire the phenotype of effector memory T cells (56, 57). Such T cells are more resistant to tolerance induction. Therefore, it is important that we employed DST-CYP before T cell depleting mAbs because dividing T cells driven by specific antigens have been shown to be more sensitive to CYP compared to T cells undergoing LIP (58). Thus, DST-CYP before the application of T cell depleting mAbs reduces the overall donor-reactive T cells and prevents the enrichment of such host T cells in LIP post-T cell depletion and BMT.

Although using T cell depleting mAb for chimerism induction in NOD mice is not new, T cell depletion was mainly used before and shortly after BMT with irradiation and/or costimulation blockade (26, 28, 30, 32, 36–38). We employed extended T cell depletion post-BMT as it provides a prolonged window for the development of donor hematopoietic cells and the education of both donor and recipient T cells. Indeed, the ability to induce chimerism was lost if fewer doses of T cell depleting mAbs were included in the triple antibody protocol, and chimerism was not rescued by adding extra NK depleting Abs or BMC (Table S2 in Supplementary Material). In addition, CYP is a cytoreductive reagent that not only reduces T cells in the periphery but also decreases immature thymocytes in the thymus (59). The combination of CYP and sufficient T cell depleting mAbs further postpones the recovery of the host T cell repertoire.

Regulatory T cells have been shown to be important for chimerism induction (60). Although they are as sensitive to DST, CYP (61), and T cell depleting antibodies (56) as conventional T cells, Tregs might still play an important role for generating chimerism in our protocol. Specific destruction of donor reactive T cells is essential for infused donor-specific Tregs to prolong graft survival (43). In addition, donor-specific Tregs can be induced during the process of chimerism induction and exert immune regulatory function (62). In fact, using our protocol, the frequency of host Tregs did increase early after BMT despite being reduced in absolute number (Figure S2 in Supplementary Material). Although we surmise that the need for recipient Tregs may depend on the efficacy of T cell depletion, this hypothesis has yet to be tested in detail.

By using the DST-CYP triple antibody protocol, we induced chimerism in NOD mice with two different fully MHC and multiple minor antigen mismatched donors, achieving a very high level of chimerism. As the induction of complete chimerism requires a more complex and intensive conditioning protocol, it was surprising that we established complete chimerism in NOD mice with such a simplified conditioning protocol without the help of irradiation, costimulation blockade, nor mega doses of BMC. By using C3H donors, induction of chimerism seemed to be easier, as the inclusion of anti-CD90 mAbs was unnecessary (Figure 2B); however, chimerism was less stable.

Some C3H→NOD mice were found sick at around 6–11 weeks post-BMT (Figures 2B,G). The morbidity in these mice was characterized as acute weight loss, hunched posture, and paleness but without skin lesions or signs of diarrhea (data not shown), which could be due to the toxicity of the conditioning regimen, GVHD, or the failure of bone marrow engraftment. In contrast, almost all recipients given FVB bone marrow and triple antibodies became stable full chimeras and remained healthy for at least 20 weeks post-BMT. In addition, body weight for FVB→NOD chimeric mice steadily increased (Figure 4A). Considering that FVB→NOD mice remained healthy, the morbidity in some C3H→NOD chimeras was unlikely to be due to the conditioning regimen.

Graft-versus-host disease has been associated with the presence of passenger T cells in the BM (63). However, passenger T cells in BM would be expected to be targeted by the anti-T cell mAbs used in our protocol with C3H donors. In addition, the inclusion of CYP and T cell depleting mAbs might help prevent GVHD, as both CYP (64, 65) and antithymocyte globulin (66) are effective for GVHD prophylaxis in the clinic. Moreover, GVHD can be avoided in patients with full chimerism with HLA mismatched donor cells (67). With the anti-CD4/8/90 conditioning regimen, stable FVB→NOD (Figure 3A) and C3H→NOD chimeras tended to be complete rather than mixed chimeras, with full donor T cell chimerism (Figure 3B). All of these chimeras were free of signs of GVHD. Therefore, complete donor chimeras induced by using a robust T cell depletion protocol can be GVHD free.

Another concern is the potential that complete chimeras will have some immunodeficiency. Though the rejection of skin graft from a third party that is MHC mismatched to either NOD or BMC donor is commonly used for determining immuno-competence of chimeras, it is not a stringent test as the natural frequency of T cells against allo-MHC is high. We showed here that FVB→NOD chimeras were able to reject skin from an MHC matched B6-g7 donor and produced antibodies in response to OVA immunization, although the rejection was delayed and the titer of anti-OVA Abs was lower when compared to otherwise much younger naive NOD mice. However, it is unclear that this would be a substantial issue, as patients with full chimerism with HLA mismatched donor cells have appeared fully immunocompetent in other studies (67, 68). On the other hand, complete chimerism is arguably favorable in autoimmune recipients, as this would more fully eliminate the cells respon-sible for autoimmunity. We showed here that stable NOD chimeras were free of autoimmune diabetes without substantial insulitis (Figure 4D).

Albeit chimerism was induced with 100% success at 4 weeks post-BMT using the triple antibody protocol, not all the chimeras maintained stable chimerism. Similar to the C3H→NOD chimeras treated with the duo antibody protocol, a slow decline of chimerism in NOD mice is common in the literature. In contrast, unstable chimeras in our study lost their high-level chimerism acutely, which has not been reported previously. On the one hand, transient mixed chimerism has been shown to be invaluable for induction of operational tolerance in allogeneic organ transplantation (41). Here, we also provide a new strategy for inducing transient high-level chimerism without using irradiation and costimulation blockade. Although donor-specific tolerance has not been tested in transient chimeras, these NOD recipients never developed hyperglycemia (Figure 4A). Whether or not such transient chimerism can be used for resolving autoimmunity remains unknown. On the other hand, by investigating the difference in early chimerism levels between stable and unstable chimeras, we found that a lower early overall level of chimerism and a lower donor T cell level were both significantly associated with the instability of long-term chimerism. Though other groups have recorded donor cell levels starting from 2 weeks post-BMT, none have reported at these earlier time points. Closely monitoring chimerism in this early window after BMT might give us some hints for the important events and cell subsets for stable chimerism induction. It also provides an opportunity for early intervention, and thus better more personalized conditioning protocols.

Although we have successfully eliminated the use of irradiation, anti-CD40L mAbs and rapamycin in our current protocol, and avoided a mega dose of BMC, there are some limitations in our approach. First, we still employed chemotherapeutic drugs, CYP and BUS, which could be improved by examining the efficacy of lower doses or replacement with other bioreagents. For instance, BUS could be replaced with anti-c-kit and anti-CD47 mAbs for creating a niche in host bone marrow (69) without radiation or chemotherapy. Second, anti-CD4/8/90 mAbs are not available for use in humans. Replacement of anti-CD4/8/90 mAbs with ATG, anti-CD52 mAbs (alemtuzumab), or other T cell depleting antibodies might be required for better T cell depletion. Third, the LIP of T cells has been associated with the development of alternative forms of autoimmunity in humans, such as in patients with multiple sclerosis who frequently develop thyroid autoimmunity post-anti-CD52 (70). Whether or not this would be the case after allogeneic BMT remains unclear. Fourth, the preferential sparing of donor CD8 T cells was associated with stable chimerism in FVB→NOD chimeras. Although this is not clinically relevant, giving donor CD8 T cells (13) or other donor cells (67) that facilitate BMC engraftment is clinically feasible. Fifth, although chimerism could be induced in 100% of naive NOD mice that were not yet diabetic using the triple antibody protocol, chimerism in some mice was not stable. Further adjustment of the current protocol or early intervention is needed to improve the chances of stable chimerism. Finally, chimerism induction in spontaneously diabetic NOD mice is more challenging (15). Whether chimerism could be generated in diabetic NOD mice with our current protocol remains to be examined.

Thus far, we achieved high-level chimerism and transplant tolerance in tolerance induction resistant NOD recipients given clinically feasible amount of donor BMC, *via* robust T cell depletion through the combination of DST-CYP and T cell depleting mAbs without the need for irradiation, costimulation blockade, and rapamycin. This protocol is, to our knowledge, the most clinically feasible to have achieved fully allogeneic mixed chimerism in NOD mice. Furthermore, unlike our previous protocol that successfully generated stable mixed chimerism in NOD mice (38), the current protocol induced robust donor-specific tolerance as evidenced by the acceptance of the most immunogenic donor tissue graft, skin. Achieving such a complete state of tolerance is likely to be even more critical in humans, where infectious agents have the potential to trigger heterologous immunity and graft rejection (71, 72). Lastly, our data point out the importance of the early window post-BMT for developing successful personalized chimerism induction protocols. We provided here a way to induce stable or transient chimerism by maximizing T cell depletion. Transient chimerism is frequently observed in combined kidney and hematopoietic stem cell transplantation (73). Our findings open the possibility of identifying early-on those patients that might lose their chimerism at later time points. Intervening to increase the stability of chimerism can be anticipated to reduce the possibility of organ/tissue rejection in these selected patients.

## Ethics Statement

This study was carried out in accordance with the recommendations of the guidelines of the Canadian Council on Animal Care. The protocol was approved by the Animal Care and Use Committees of the University of Alberta.

## Author Contributions

JL designed and performed experiments, analyzed data, and wrote the manuscript; CA designed experiments, analyzed data, and wrote the manuscript; WC designed and performed experiments, analyzed data and edited the manuscript; LB generated reagents and edited the manuscript.

## Conflict of Interest Statement

Author LB was employed by company Bioceros (Netherlands). All other authors declare no competing interests. The reviewer CL and handling Editor declared their shared affiliation.

## Abbreviations

anti-CD40L, anti-CD40 ligand; BMC, bone marrow cells; BMT, bone marrow transplantation; CYP, cyclophosphamide; DST, donor-specific transfusion; PBL, peripheral blood lymphocytes; GVHD, graft-versus-host disease; NOD, non-obese diabetic; TBI, total body irradiation; MHC, major histocompatability complex; MiHA, minor histocompatability antigen; PBS, phosphate-buffered saline; i.p., intraperitoneal.
