# Supplementary figures and images for: Stability of Chimerism in Non-Obese Diabetic Mice Achieved By Rapid T Cell Depletion Is Associated With High Levels of Donor Cells Very Early After Transplant
Source: Front Immunol. 2018 Apr 24;9:837. doi: 10.3389/fimmu.2018.00837 (PMC5928230; doi:10.3389/fimmu.2018.00837)

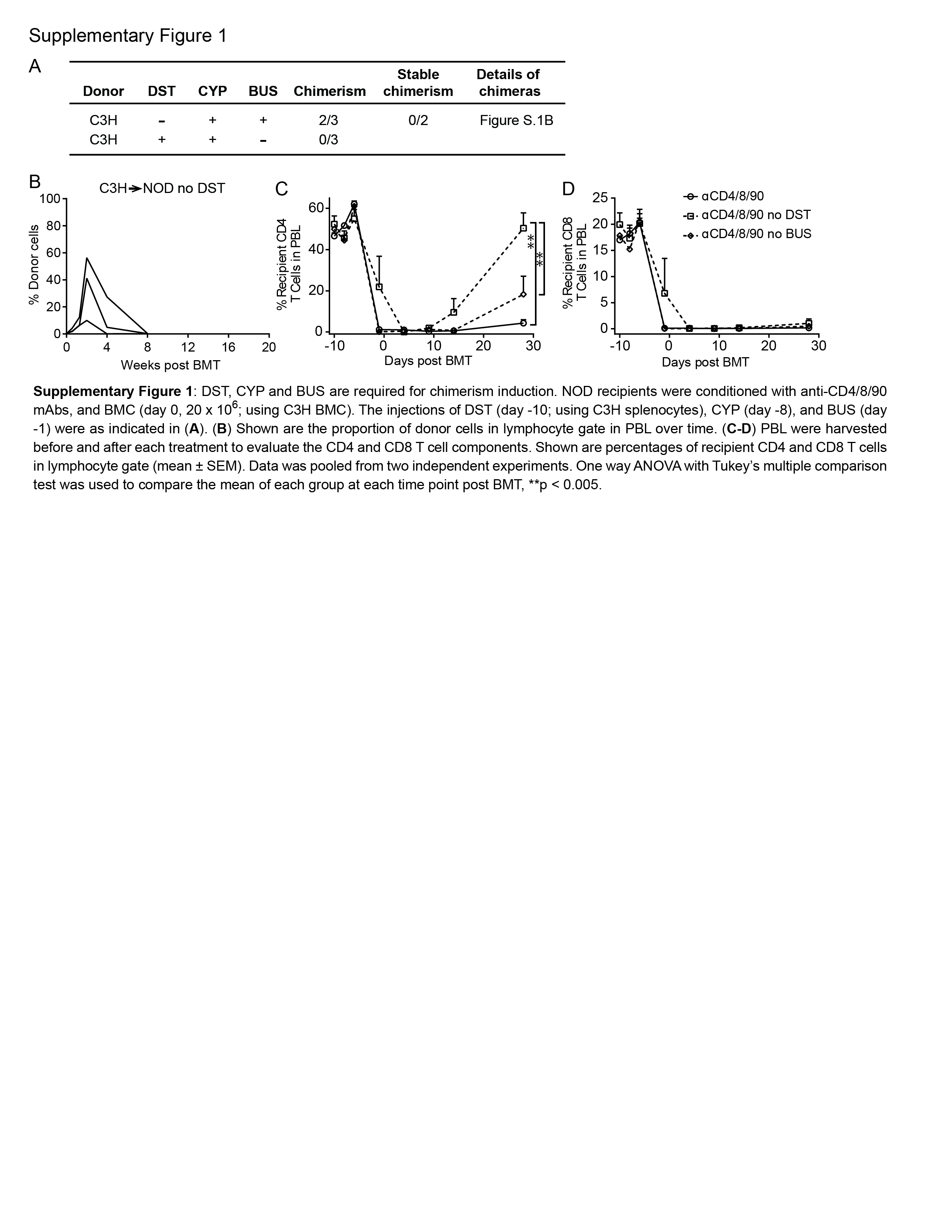

Supplement: Supplementary file 1 [file image_1.TIF]

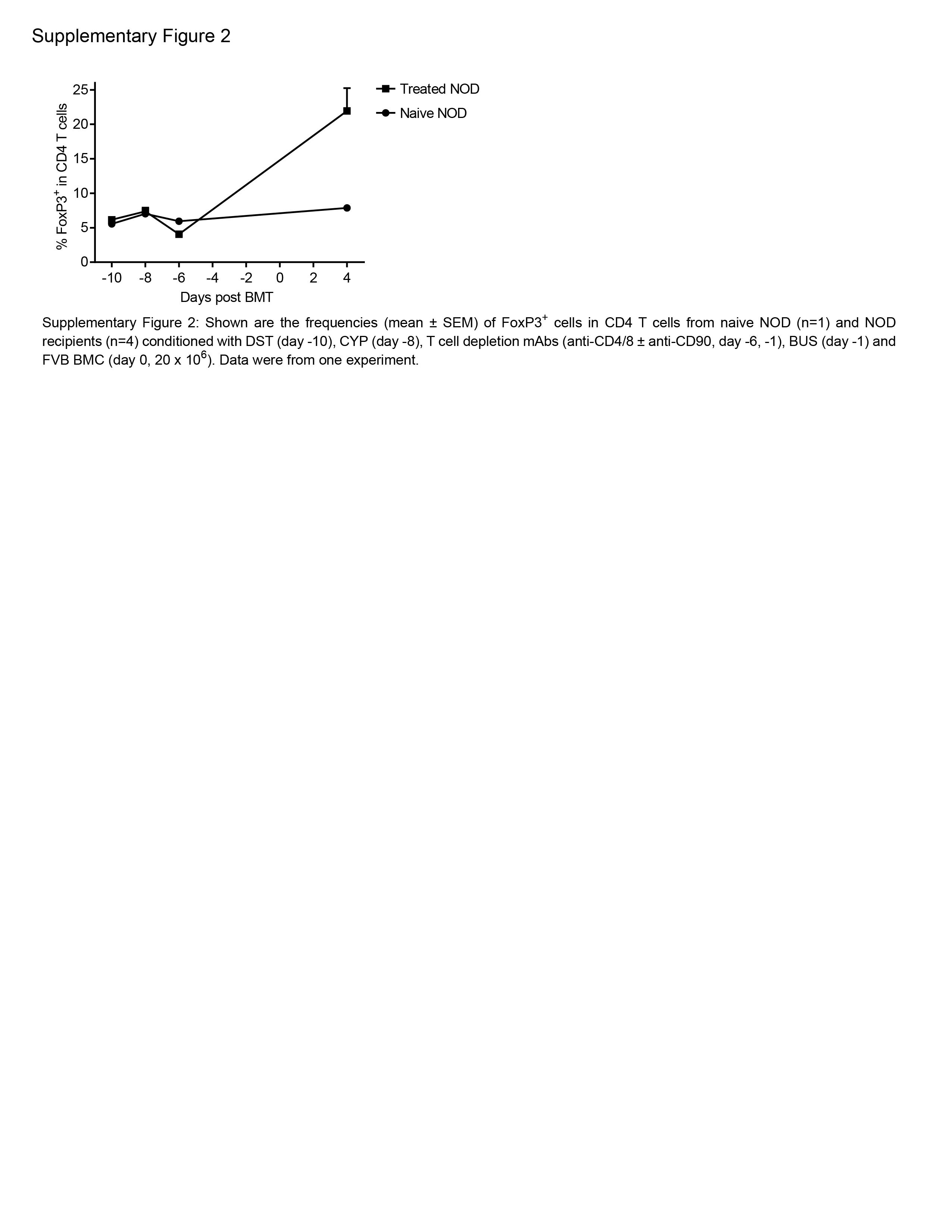

Supplement: Supplementary file 2 [file image_2.TIF]
