# Supplementary material for: Stability of Chimerism in Non-Obese Diabetic Mice Achieved By Rapid T Cell Depletion Is Associated With High Levels of Donor Cells Very Early After Transplant
Source: Front Immunol. 2018 Apr 24;9:837. doi: 10.3389/fimmu.2018.00837 (PMC5928230; doi:10.3389/fimmu.2018.00837)
Supplement: Supplementary file 3 [file table_1.PDF]

Supplementary Table 1

Haplotype of mice used in chimerism induction

Mouse	MHC-I			MHC-II	
	H2-K	H2-D	H2-L	I-A	I-E
NOD	d	b	-	g7	-
C3H	k	k	-	k	k
FVB	q	q	q	q	-
B6-g7	d	b	-	g7	-
