# Supplementary material for: Stability of Chimerism in Non-Obese Diabetic Mice Achieved By Rapid T Cell Depletion Is Associated With High Levels of Donor Cells Very Early After Transplant
Source: Front Immunol. 2018 Apr 24;9:837. doi: 10.3389/fimmu.2018.00837 (PMC5928230; doi:10.3389/fimmu.2018.00837)
Supplement: Supplementary file 4 [file table_2.PDF]

Supplementary Table 2

| Donor | αCD4/8/90 | Donor BM ($x 10^{-6}$) | chimerism |
|---|---|---|---|
| FVB | x2 | 20 or 30 | 0/4 |
| FVB or C3H | x3 | 10 or 20 | 0/4 |
| C3H | x4 | (10 or 20)x2‡ | 0/2 |
| C3H | x5 | 20 | 3/3 |
| FVB | x9† | 20 | 2/2 |

Extended T cell depletion post BMT is needed for chimerism induction in naïve NOD mice. NOD recipients were conditioned with DST (day -10), CYP (day -8), anti-CD4/8/90 (day -6 and every 5 days afterwards; x2, x3, x4, x5, x9 indicate 2, 3, 4, 5, 9 doses; † mice were injected on day -6, -1, 4, 9, 15, 21, 27, 34, 41), anti-AsGM1 (day -6 and day -1, 20 μL), BUS (day -1) and BMC (day 0; 10, 20, or 30 x $10^6$; ‡ mice were infused with one more dose of BMC on day 9; cells were from the same donor strain for DST). Chimerism levels were determined at 4 weeks post BMT.
